# Atorvastatin-induced pancreatitis

**DOI:** 10.4103/0253-7613.70400

**Published:** 2010-10

**Authors:** Samir Prajapati, Samidh Shah, Chetna Desai, Mira Desai, R.K. Dikshit

**Affiliations:** Department of Pharmacology, B. J. Medical College, Ahmedabad, India

**Keywords:** Adverse drug reaction, atorvastatin, pancreatitis

## Abstract

Drugs account for 1–2% of all cases of pancreatitis. A 58-year-old man was prescribed atorvastatin 10 mg for 6 months for hyperlipidemia. He developed acute abdominal pain and vomiting with epigastric tenderness. Serum lipase and CT scan of the patient suggested the presence of acute pancreatitis. The patient was hospitalized; atorvastatin was stopped and treated symptomatically. He recovered completely within 10 days of drug withdrawal. The causality of the adverse drug reaction according to Naranjo and WHO-UMC Scale was probable. The exact mechanism of pancreatitis due to atorvastatin is not known. It may be a class effect of HMG CoA reductase inhibitors as it had been reported with other statins too. The definite causal relationship is difficult to establish, as rechallenge with the suspected drug was not done due to ethical consideration.

## Introduction

Statins are commonly used for the treatment of hyperlipidemia. Atorvastatin is a frequently prescribed agent among them. They are generally considered safe, except for the risk of myopathy and liver damage. Recently, some cases of statin-induced pancreatitis have been reported.[1] In this study, we report a case of pancreatitis probably induced by atorvastatin.

## Case Report

**Figure 1:**
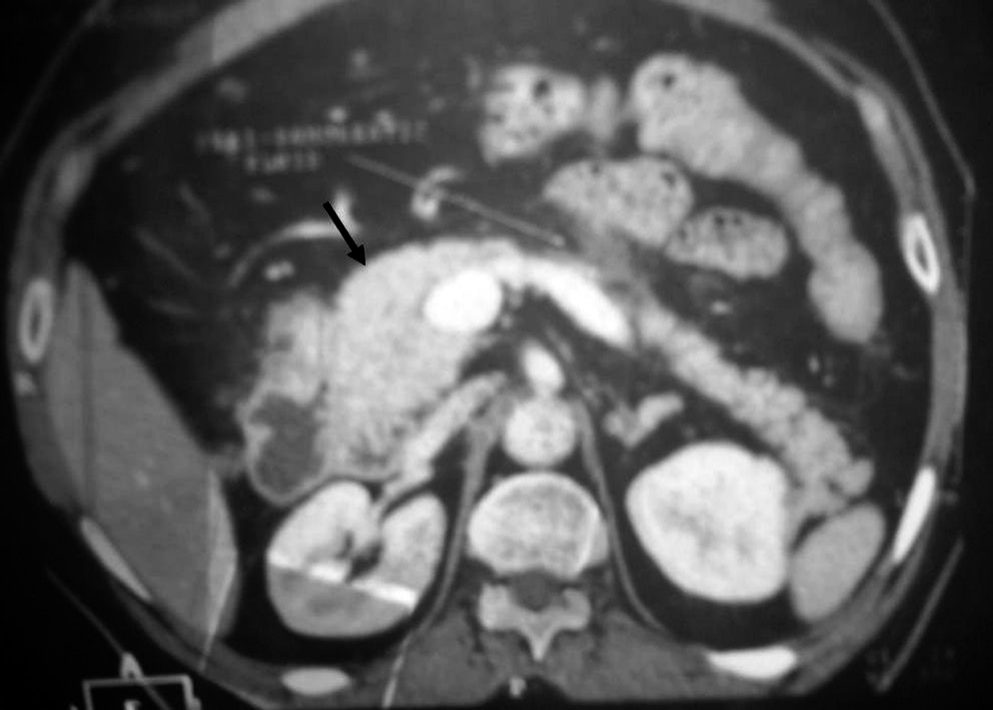
CT scan shows head of the inflamed pancreas

A 58-year-old man presented with severe vomiting and abdominal pain. There was no history of trauma or addiction to alcohol. There was no family history of pancreatitis. The patient was a known case of hyperlipidemia and was prescribed tablet atorvastatin 10 mg for last 6 months. He was also taking ferrous sulfate for anemia and ranitidine for dyspepsia for last 2 years. Physical examination revealed mild abdominal distention with severe epigastric tenderness. Laboratory investigations revealed serum lipase (137 IU/dL, normal range: 16–63 IU/dL) and serum amylase (501 IU/dL, normal range: 0–85 IU/dL] CT scan showed inflammation of the head of pancreas [Figure 1]. Ultrasonography (USG) of abdomen, magnetic resonance cholangiopancreatography (MRCP) and esophagoscopy were normal. The patient was diagnosed as a case of acute pancreatitis and hospitalized. Atorvastatin was stopped, and patient was treated symptomatically. Patient improved (serum lipase: 48 IU/dL on discharge) and was discharged from the hospital after 10 days.

Causality assessment of the adverse drug event (ADE) was carried out using WHO–UMC criteria and Naranjo’s Scale. In this case, the patients improved on dechallenge (withdrawal of the drug) and there were no other confounding factors that could have caused this ADE. Hence, the ADE was probably caused by atorvastatin (WHO–UMC criteria: Probable; Naranjo’s Score: 7, probable). The reaction was severe in nature (modified Hartwig and Siegel’s Scale),[2] and it was not preventable (modified Schumock and Thornton criteria).[3]

## Discussion

Drugs account for 1–2% of all cases of pancreatitis.[4] Numerous (more than 260) drugs, including statins have been implicated in the pathogenesis of acute pancreatitis.[5] Drug-induced pancreatitis is a clinical entity for which causal relationship is difficult to establish. Most information comes through individual case reports. In the present case, USG, CT scan, and MRCP ruled out the possibility of a surgical cause of pancreatitis. Patient was nonalcoholic, and there was no past history of pancreatic disease. Serum calcium and triglycerides were within normal range. Patient improved after dechallenge (withdrawal of the suspected drug). These factors raise a suspicion about causal relationship between atorvastatin and pancreatitis in this patient.

The exact mechanism of pancreatitis due to atorvastatin is not known. It may be a class effect as it is reported with other statins. The duration of statin treatment until the onset of pancreatitis is also variable, occurring within the first day of therapy in some cases and after several months in others.[6] Reports of outcome after rechallenge with the same or other statins are lacking. Therefore, a causal relation cannot be established with certainty, although in one previous report, the patient had tolerated pravastatin after developing atorvastatin-induced pancreatitis.[7] Statins are frequently prescribed for a variety of indications such as diabetes mellitus, ischemic heart diseases, cerebrovascular diseases, etc. Hence, the prescriber should, therefore, be aware of this ADR while evaluating patients suffering from pancreatitis. They also need to be aware that this might be a class effect of statins and hence prescribing another statin may also not be safe. Further research is needed to identify the exact mechanism of pancreatic injury induced by statins.
